# Comprehensive Bioinformatics Identifies Key microRNA Players in ATG7-Deficient Lung Fibroblasts

**DOI:** 10.3390/ijms21114126

**Published:** 2020-06-09

**Authors:** Stevan D. Stojanović, Maximilian Fuchs, Jan Fiedler, Ke Xiao, Anna Meinecke, Annette Just, Andreas Pich, Thomas Thum, Meik Kunz

**Affiliations:** 1 Institute of Molecular and Translational Therapeutic Strategies (IMTTS), Hannover Medical School, 30625 Hannover, Germany; Stojanovic.Stevan@mh-hannover.de (S.D.S.); Fiedler.Jan@mh-hannover.de (J.F.); Xiao.Ke@mh-hannover.de (K.X.); Meinecke.Anna@mh-hannover.de (A.M.); Just.Annette@mh-hannover.de (A.J.); 2 Chair of Medical Informatics, Friedrich-Alexander University of Erlangen-Nürnberg, 91058 Erlangen, Germany; maximilian.fuchs@fau.de; 3 Functional Genomics and Systems Biology Group, Department of Bioinformatics, University of Würzburg, Würzburg 97074, Germany; 4 Institute of Toxicology and Core Unit Proteomics, Hannover Medical School, 30625 Hannover, Germany; Pich.Andreas@mh-hannover.de; 5 REBIRTH Center for Translational Regenerative Medicine, Hannover Medical School, 30625 Hannover, Germany

**Keywords:** bioinformatics, miR, proteomics, functional network analysis, senescence, lung fibrosis, autophagy

## Abstract

Background: Deficient autophagy has been recently implicated as a driver of pulmonary fibrosis, yet bioinformatics approaches to study this cellular process are lacking. Autophagy-related 5 and 7 (ATG5/ATG7) are critical elements of macro-autophagy. However, an alternative ATG5/ATG7-independent macro-autophagy pathway was recently discovered, its regulation being unknown. Using a bioinformatics proteome profiling analysis of ATG7-deficient human fibroblasts, we aimed to identify key microRNA (miR) regulators in autophagy. Method: We have generated ATG7-knockout MRC-5 fibroblasts and performed mass spectrometry to generate a large-scale proteomics dataset. We further quantified the interactions between various proteins combining bioinformatics molecular network reconstruction and functional enrichment analysis. The predicted key regulatory miRs were validated via quantitative polymerase chain reaction. Results: The functional enrichment analysis of the 26 deregulated proteins showed decreased cellular trafficking, increased mitophagy and senescence as the major overarching processes in ATG7-deficient lung fibroblasts. The 26 proteins reconstitute a protein interactome of 46 nodes and miR-regulated interactome of 834 nodes. The miR network shows three functional cluster modules around miR-16-5p, miR-17-5p and let-7a-5p related to multiple deregulated proteins. Confirming these results in a biological setting, serially passaged wild-type and autophagy-deficient fibroblasts displayed senescence-dependent expression profiles of miR-16-5p and miR-17-5p. Conclusions: We have developed a bioinformatics proteome profiling approach that successfully identifies biologically relevant miR regulators from a proteomics dataset of the ATG-7-deficient milieu in lung fibroblasts, and thus may be used to elucidate key molecular players in complex fibrotic pathological processes. The approach is not limited to a specific cell-type and disease, thus highlighting its high relevance in proteome and non-coding RNA research.

## 1. Introduction

Interstitial lung diseases are a complex group of alveolar pathologies featuring aberrant cell proliferation, inflammation and fibrosis [1]. Amongst several categories of interstitial lung diseases, idiopathic pulmonary fibrosis (IPF) is the most common subtype [1]. Despite its relative frequency, which is the highest amongst the older population, IPF is not fully understood. As a result, the disease is characterized by unrelenting irreversible chronic fibrosis and poor median survival (2–4 years) [2]. Furthermore, approved therapeutic options are limited [2].

Autophagy is emerging as a critical pathophysiological player in pulmonary fibrosis [3,4]. This process is defined as the proteolytic degradation of cytosolic cellular components [5]. Its subtype is macro-autophagy, which involves the autophagosomal-mediated catabolism of cellular components [5]. Autophagy related 7 (ATG7) is a key component of conventional macro-autophagy, allowing the conversion of microtubule-associated proteins 1A/1B light chain 3B (LC3BI) to LC3BII via phosphatidylethanolamine conjugation. LC3BII is integrated into the phagophore membrane with the help of the ATG12-ATG5:ATG16L1 complex, whose creation is also enabled by ATG7, leading to autophagosome maturation [6]. Mature autophagosomes bind to lysosomes, allowing the degradation of proteins and organelles [6].

Macro-autophagy declines with aging [7] and IPF [8], possibly accounting for the predisposition of older individuals to lung fibrosis. On a functional level, lack of ATG5-derived macro-autophagy caused pro-fibrotic changes in alveolar epithelial cells [9]. Impaired conventional ATG7-dependent macro-autophagy in lung endothelium increased susceptibility to bleomycin-induced fibrosis in mice [10]. Fibroblasts of IPF patients cannot engage autophagy under stress [11]. This caused vimentin and collagen accumulation due to a lack of catabolism and activation of pro-fibrotic activity in these cells [11].

However, a non-conventional autophagosomal ATG5/7-independent and thus alternative macro-autophagy pathway (ATG5/7alt; ATG5/ATG7-independent alternative macro-autophagy pathway) has been recently discovered in mouse embryonic fibroblasts [12]. This autophagosomal pathway was not involved with LC3B conversion, and its proposed role was mitophagy [12]. Interestingly, ATG7 loss of function has so far not been studied in lung fibroblasts, which are the principal producers of the extracellular matrix in lung fibrosis [13,14,15,16,17]. Moreover, the regulation of ATG5/7alt is unclear.

Autophagy processes are actively regulated at the post-transcriptional level through microRNA (miR) [18]. miRs typically affect proteins’ expression by inhibiting their mRNA transcript translation [19]. Additionally, miR regulators are often cell-type specific [20,21]. The identification of key autophagy-related miR can introduce tools to fine-tune autophagy on a pre-translational level and in a cell-type specific manner, thus providing a potential therapeutic strategy for the treatment of IPF. Therefore, we have set out to identify miR-regulators of ATG5/7alt proteome profiling in human lung fibroblasts using a comprehensive bioinformatics analysis approach.

## 2. Results

### 2.1. ATG7-Knockout Disables LC3B Traffic in Endothelial Cells and Fibroblasts

**Figure 1:**
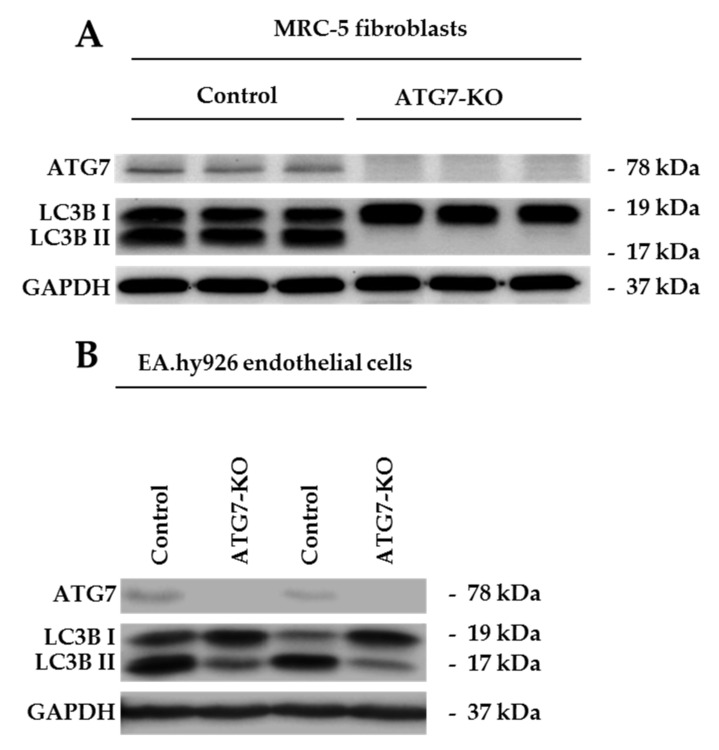
Western Blot analysis of the ATG7-knockout (ATG7-KO) in endothelial cells and fibroblasts. (**A**) Western Blot of ATG7, LC3BI and II in control versus ATG7-KO-MRC-5 fibroblasts (**B**) Western Blot of ATG7, LC3BI and II in control versus ATG7-KO in EA.hy926 endothelial cells.

To initiate a switch towards ATG7-independent autophagy, the key autophagosomal maturation protein ATG7 was targeted via Clustered regularly interspaced short palindromic repeats (CRISPR)-Cas9 mediated deletion in two different cell types: MRC-5 fibroblasts and EA.hy926 endothelial cells. This gene-editing strategy caused a depletion of ATG7 in both *in vitro* models (Figure 1A,B). Conventional macro-autophagy inhibition was confirmed on a functional level for both cell types through impaired LC3BI to LC3BII conversion in ATG7-CRIPSR groups (Figure 1A,B). Therefore, EA.hy926-ATG7-KO (ATG7-knockout) and MRC-5-ATG7-KO have a deficient conventional macro-autophagy at baseline levels.

### 2.2. Autophagosomal Generation is Hampered in ATG7-Knockout Endothelial Cells, but not in Lung Fibroblasts

**Figure 2:**
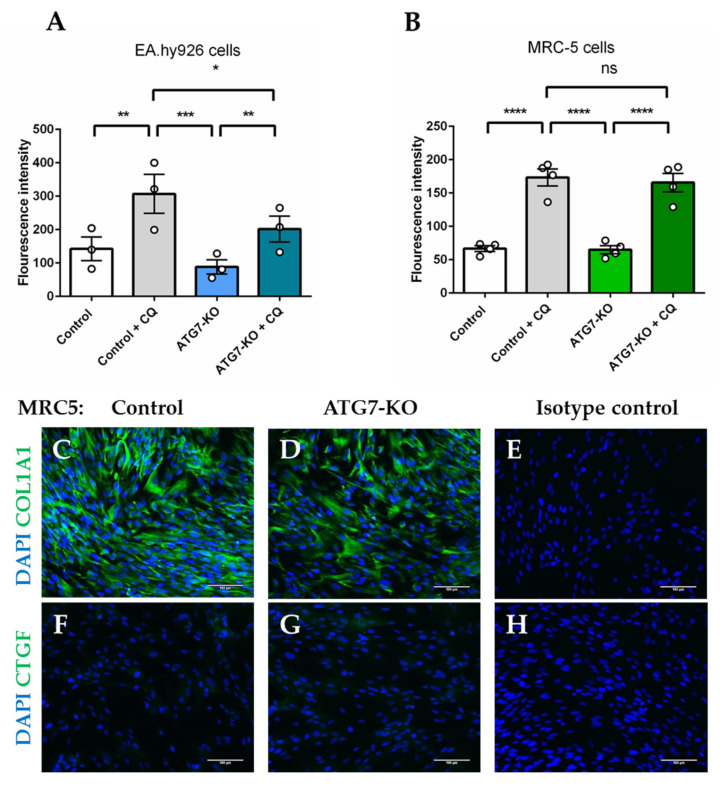
Autophagosomal inhibition of the ATG7-knockout (ATG7-KO) in endothelial cells and fibroblasts. (**A**) Autophagy flux Fluorescence-activated cell sorting (FACS) measurements of control and ATG7-KO-EA.hy926 endothelial cells (**B**) Autophagy flux Fluorescence-activated cell sorting (FACS) measurements of control and ATG7-KO-MRC-5 fibroblasts cells (**C**–**E**) COL1A1 immunofluorescence of control and ATG7-KO-MRC-5 cells (F-H) CTGF immunofluorescence of control and ATG7-KO-MRC-5. Scale bar represents 100 µm. * *p* < 0.05, ** *p* < 0.01, *** *p* < 0.001, **** *p* < 0.0001, 2-way ANOVA.

MRC-5 fibroblasts and EA.hy926 belonging to control or ATG7-knockout (ATG7-KO) groups were exposed to autophagy-inhibiting conditions (serum-starvation with and without chloroquine (CQ) treatment). Serum-starved control and ATG7-KO-EA.hy926 cells had similar autophagosome accumulation at baseline. However, upon autophagosomal inhibition with CQ, EA.hy926 ATG7-KO showed significantly lower levels of autophagosomal fluorescence than controls treated with CQ, failing to fully maintain the autophagic flux (Figure 2A). On the other hand, both control and ATG7-KO-MRC-5 retained the ability to generate autophagosomes within the same conditions (Figure 2B). Furthermore, ATG7-KO in fibroblasts did not cause Collagen, type I, alpha 1 (COL1A1) accumulation (Figure 2C–E) or Connective tissue growth factor (CTGF) activation (Figure 2F–H), which are features of active matrix-producing fibroblasts [22,23]. This cell-type specific response implied that MRC-5 fibroblasts activate ATG7-independent autophagy, which is sufficient to prevent pro-fibrotic features and is a suitable model of studying miR regulators of ATG5/7alt.

### 2.3. LC-MS Shows a Signature of ATG5/7-Independent Autophagy in ATG7-Knockout Lung Fibroblasts

**Figure 3:**
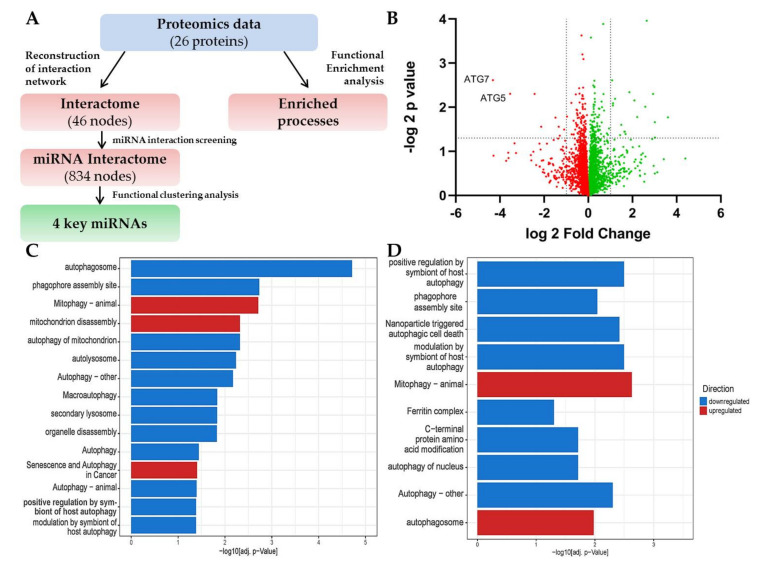
(**A**) Workflow of our comprehensive bioinformatics proteome profiling approach. (**B**) Volcano plot representation of control and ATG7 MRC-5 LS-MS proteomic data. *n* = 3, *t*-test. (**C**) Enrichment analysis of both downregulated and upregulated proteins, mitophagy and senescence processes highlighted in red. (**D**) Upregulated (red) and downregulated (blue) processes in ATG7-KO-MRC-5 fibroblasts.

Utilizing the opportunity to study actively regulated processes in deficient autophagy, we have performed Liquid chromatography–mass spectrometry (LC-MS) on control and ATG7-KO-MRC-5 fibroblasts and designed an integrated bioinformatics prediction pipeline to enable comprehensive data analysis (Figure 3A). LC-MS generated an unbiased proteomics set of 107 upregulated and 97 downregulated proteins (*p* < 0.05; Figure 3B; Table S1). ATG7, but also ATG5, were amongst the lowest expressed proteins in the dataset, confirming the CRISPR-knockout. The initial functional analysis of the 26 significantly regulated proteins (*p* < 0.05; logFC < –1/> 1; Table S1) revealed the significant involvement of autophagy perturbations, which involved mitophagy and senescence (highlighted in red, Figure 3C). Further analysis of only downregulated proteins (Table S1) showed an enrichment of processes associated with conventional macro-autophagy (in blue, Figure 3D). Amongst the upregulated factors, there were several proteins involved with mitophagy (Calcium-binding and coiled-coil domain-containing protein 2 (CALCOCO2), Sequestosome-1 (SQSTM1), Gamma-aminobutyric acid receptor-associated protein-like 2 (GABARAPL2), Table S1), the mitophagic and autophagosome processes being enriched (in red, Figure 3D), confirming the principal role of ATG5/7alt in autophagosomal-mediated mitophagy. Therefore, ATG7-KO MRC-5 fibroblasts had an active ATG5/7alt, which was functionally mitophagic. Moreover, such a metabolic switch predicted the development of senescence, implicating this cellular fate as the phenotypical outcome of the aforementioned molecular interactions.

### 2.4. Bioinformatics Approach Predicts miR-16-5p, miR-17-5p, let-7a-5p and miR-93-5p as Key Regulators of ATG5/7alt with Biological Relevance in Senescence

**Figure 4:**
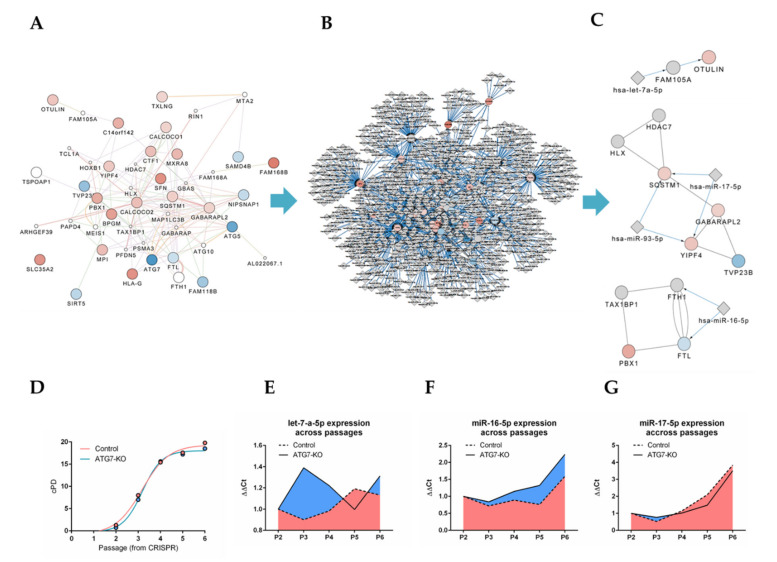
Comprehensive bioinformatics proteome profiling analysis. (**A**) Significantly deregulated protein interaction network. Red spheres indicate upregulated proteins, blue indicate downregulated proteins. White spheres are inserted predicted interaction partners. (**B**) miR-regulated network analysis of all possible miR regulators of network proteins. (**C**) The functional cluster analysis identified three functional cluster modules between miR-16-5p, miR-17-5p, let-7a-5p, miR-93-5p and their targets related to the network proteins. (**D**) Cumulative population doubling curve (cPD) of control and ATG7-KO-MRC-5 fibroblasts. (**E**–**G**) Relative expression levels (∆∆Ct) of let-7a-5p, miR-16-5p and miR-17-5p normalized to Small nucleolar RNA SNORD48 (RNU-48). *P* signifies passage number.

A deep *in silico* analysis with a strict cut-off of *p* < 0.05 revealed a network of 46 proteins with physical and functional interactions, implying an orchestrated pathway organization (Figure 4A). Next, interactome proteins were overlapped with their miR regulators, which resulted in a miR-regulated network of 834 nodes (Figure 4B), which was narrowed down to three well-connected functional clusters around four critical miR candidates (miR-16-5p, miR-17-5p, let-7a-5p and miR-93-5p) linked to the mRNA of multiple deregulated proteins (Figure 4C). Since cellular senescence was predicted as a phenotypical outcome of the ATG7-knockout, we generated a replicative senescence timeline of control and ATG7-KO-MRC-5 (Figure 4D). We then proceeded to measure miR-16-5p, miR-17-5p and let-7a-5p levels across passages of control and ATG7 fibroblasts. miR-93-5p was excluded due to primer unavailability. While let-7a-5p showed a complex regulation pattern between two groups (Figure 4E), miR-16-5p (Figure 4F) and miR-17-5p (Figure 4G) indeed followed a passage-dependent expression trend in the cellular aging of both wild type and ATG7-KO fibroblasts, indicating the correct functional outcome of our functional *in silico* predictions. Thus, we validated that our bioinformatics proteome profiling approach correctly derives miRs involved with ATG5/7alt-independent autophagy by predicting their phenotypical involvement in senescence.

## 3. Discussion

We have simulated a conventional autophagy deficit in endothelial cells and fibroblasts by generating ATG7-knockouts. ATG7-knockout endothelial cells could not fully recover autophagosomal generation after autophagy inhibition via CQ. On the other hand, ATG7-knockout fibroblasts fully retained the capacity to generate autophagosomes, even after CQ treatment. Therefore, while ATG7-knockout fibroblasts had impaired the conventional macro-autophagy-related LC3B conversion, they still maintained the autophagosomal generation. These findings indicate a cell-specific molecular signature of an active autophagosomal ATG5/7alt in ATG7-knockout fibroblasts, but likely not in ATG7-knockout-endothelial cells [12]. ATG5/7alt appeared to be sufficient to prevent COL1A1 buildup and fibroblast activation. Pirfenidone, one of two Food and Drug Administration (FDA)-approved drugs for IPF, provides anti-fibrotic effects partly by increasing mitophagy [24]. Given the role of ATG5/7alt in promoting mitophagy, it may be the responsible pathway for the benefits of pirfenidone, which could be directly therapeutically exploited. Speculatively, collagen buildup and fibroblast activation may occur mainly due to mitophagy defects and independently of conventional macro-autophagy, but this requires future in-depth study.

**Figure 5:**
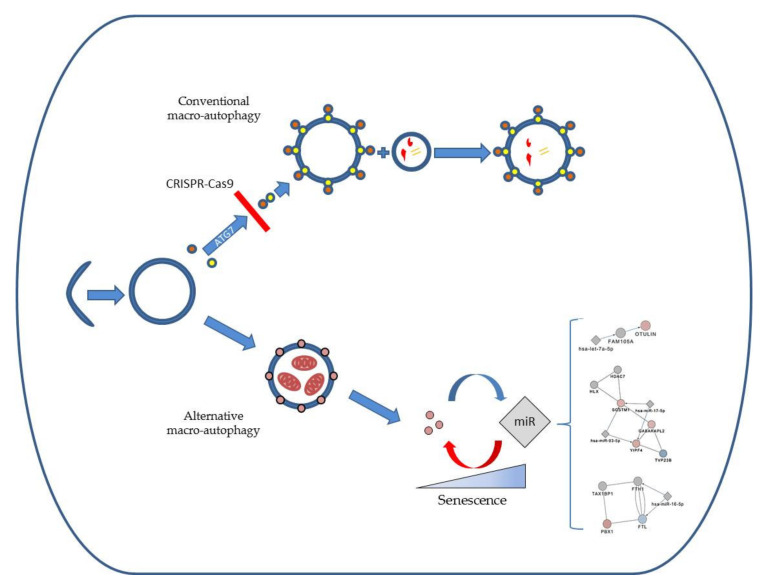
Schematic overview of the ATG5/7 macro-autophagy regulation derived from our profiling analysis.

To facilitate the understanding of ATG5/7alt regulation via miRs, we have performed unbiased LC-MS on control and ATG7-KO fibroblasts to generate a proteomic dataset of proteins deregulated early in the process. The knockout of ATG7 enabled the study of proteins associated with ATG5/7alt. In addition to the ATG7 knockout, we observed downregulated ATG5 in our proteomics data. The exact mechanism of this phenomenon is unclear. ATG5 is usually identified as a part of the ATG12-ATG5:ATG16L1 protein complex [25]. The identification of isolated ATG5 outside of the mentioned complex was reported to be difficult, likely due to its covalent bond with ATG12 [25]. Indeed, ATG7 is important for the ATG12-ATG16L1-ATG5 complex creation [6]. ATG7 knockout thus likely caused a downstream loss of the entire complex. This may be an explanation for the concomitant downregulation of ATG5 after ATG7 knockout. Of note, ATG7 may have functions that are not immediately related to autophagy [26], possibly affecting the proteomics data expression in the knockout. However, our bioinformatics approach showed that the most highly deregulated proteins were dominantly associated with autophagy processes. We have thus proceeded to overlap these proteins with interacting proteins and their potential miR regulators and to narrow down the output to key miR candidates (miR-16-5p, miR-17-5p and let-7a-5p). miR-16-5p and miR-17-5p seemed to follow a passage-related pattern, while let-7a-5p regulation was complex in a mirrored pattern between groups. In addition, cellular senescence was the predicted functional outcome of the ATG7-knockout (Figure 5). The knockout highlighted various deregulated proteins which are key nodes in the non-conventional alternative macro-autophagy pathway.

Intriguingly, emerging evidence points to cellular senescence as an attractive target for the treatment of IPF [27]. Senescent cells have been proposed to drive several diseases [28]. This includes IPF, where senescent cells showed a pro-inflammatory and pro-fibrotic phenotype [27,29,30].

Autophagy and senescence form a dual relationship [31,32,33]. While macro-autophagy helps senescent cells to keep up with the intense metabolic demands of their pro-inflammatory metabolism [31], selective autophagy of GATA4 suppresses senescence [34]. Mitochondrial dysfunction [35] and decreased mitophagy [36,37,38] are consistent features of senescence. Our results are in line with these findings, by showing that an active ATG5/7alt pathway is sufficient to maintain replication and allows cells from both experimental groups to reach senescence in a similar manner.

A systems biology hypothesis can explain these results. The ATG-7 knockout pointed towards various deregulated proteins which are key nodes in the non-conventional alternative autophagy pathway. Next, our bioinformatics approach identified key miRs that regulate these nodes and were biologically relevant in the senescence of both experimental groups. The computational prediction indicated the possibility of a reverse feedback pathway [39] originating from upregulated SQSTM1 and GABARAPL2 proteins in autophagy. This may have caused a responsive miR-17-5p upregulation aimed at their negative regulation. A similar mechanism may be in place, albeit indirectly, for miR-16-5p and Pre-B-cell leukemia transcription factor 1 (PDX1), as PDX1 depletion is known to induce senescence [40] and impaired mitophagy [41]. Therefore, we suspect that the presence of active ATG5/7alt pathway elements in both control and knockout models caused a gradual pressure on the miR feedback circuitry, eventually tipping the balance toward miR upregulation, resulting in ATG5/7alt suppression, which in turn may have led to mitochondrial dysfunction and senescence.

## 4. Materials and Methods

### 4.1. Chemicals and Antibodies

Chloroquine was purchased from Enzo, Farmingdale, NY, USA (CYTO-ID^®^ Autophagy Detection kit, ENZ-51031-51005-CLQ). The following antibodies were used: ATG7 (Santa Cruz, TX, USA, sc-376212), COL1A1 (Sigma Aldrich, St. Louis, MO, USA, HPA008405), CTGF (Abcam, Cambridge, United Kingdom, ab6992), GAPDH (Abcam, Cambridge, United Kingdom, ab8245) and LC3B (Abcam, Cambridge, United Kingdom, ab48394).

### 4.2. Cell Culture and Genome Editing

Human fibroblasts from fetal lungs (MRC-5) and endothelial cells (EA.hy926) were originally purchased from ATCC, Gaithersburg, MD, USA. The cells were cultured in Dulbecco’s Modified Eagle Medium (Thermo Fisher, Dreieich, Germany, 11965084), supplemented with 10% FBS and 1% penicillin-streptomycin (Sigma Aldrich, St. Louis, MO, USA). To achieve serum-starvation, FBS was omitted. For CRISPR-Cas9 genome editing, we modified a previously published protocol [42]. CRISPR-based cloning was done with the plasmid (lenticrispv2) purchased from Addgene, Watertown, MA, USA (52961). sgRNA oligonucleotides for subcloning to lenticrisprv2 were acquired from Eurofins Genomics, Ebersberg, Germany. Puromycin was used for the selection process after transduction with viral particles. To achieve replicative senescence, cells were serially passaged. Cells were cultured after genome editing until they reached around 80% confluence and were then re-seeded in T75 Flasks (Sarstedt, Nümbrecht, Germany, 83.3911.002) at 3 × 10^3^ cells/cm^2^. Cumulative population doublings were calculated via the formula cPD = X+3.322*(log Y-log I), X being the cumulative population doubling of the subculture initiating the culture, Y representing the viable cell number on harvest and I being the cell number on seeding.

### 4.3. Protein Isolation and Western Blot

Total protein was obtained from cell pellets previously washed with PBS, through digestion via the lysis buffer (Bio-Rad, Hercules, CA, USA). Samples were loaded on polyacrylamide gels for electrophoresis and transferred to a nitrocellulose membrane (Bio-Rad, Hercules, CA, USA). Blocking was performed for 1 h with 5% milk solution, and membranes were incubated with the primary antibody overnight. After washing, the membranes were incubated with the Secondary IgG-HRP secondary antibody (Cell Signaling, MA, USA), ultimately being rinsed and envisioned by using the enhanced chemiluminescence (ECL) reagent (Bio-Rad, Hercules, CA, USA).

### 4.4. RNA Isolation and RT-qPCR

Total RNA was isolated using the miRNeasy Mini Kit (Qiagen, Hilden, Germany) according to the manufacturer’s instructions. The subsequent quantification and quality control were performed with the Synergy HT Reader (BioTek, Winooski, VT, USA). For miRNA quantification, specific TaqMan MiR Assays were used following the manufacturer’s protocol (Thermo Fisher, Dreieich, Germany, assay IDs: 000391 for miR-16-5p, 002308 for miR-17-5p and 000377 for let-7a-5p). MiRNAs were quantified using the ViiA™ 7 Real-Time PCR System (Life Technologies, Carlsbad, USA). MiRNA levels were normalized to the Small nucleolar RNA SNORD48 (RNU48, Thermo Fisher, Dreieich, Germany, assay ID: 001006).

### 4.5. FACS for Autophagosomal Fluorescence

For the detection of autophagy, Ea.Hy926 and MRC-5 cells were stained with CYTO-ID^®^ Green dye (Enzo, Farmingdale, NY, USA) according to the manufacturer’s instructions. In brief, the cells were incubated with CYTO-ID staining solution, and the mean fluorescence intensity for CYTO-ID^®^ Green was assessed using the guava easyCyteTM Flow Cytometer (Merck–Millipore, Temecula, CA, USA).

### 4.6. Immunofluorescence

Cells were fixed using 4% formaldehyde (Sigma Aldrich, St. Louis, MO, USA) for 15 min, rinsed with Phosphate Buffered Saline (PBS), incubated with donkey serum as blocking buffer for 30 min. After adding primary antibodies in the blocking buffer, the cells were incubated overnight in the dark, and washed and stained with secondary anti-body Alexa Flour 488 (Thermo Fisher, Dreieich Germany). The cells were then again rinsed with PBS and stained with DAPI (Thermo Fisher, Dreieich, Germany). Isotype controls had primary anti-bodies excluded from the protocol.

### 4.7. LC-MS

LC-MS was performed as described previously [43]. In short, the whole cell lysate total protein from MRC-5 fibroblast controls and ATG7-KO total protein was alkylated via acrylamide, separated on pre-casted SDS gels (Bio-Rad, Hercules, CA, USA) and incubated via the Coomassie solution for 45 min at room temperature. The gel was thereafter destained, the lanes trypsinized, and the resulting peptides were separated with a LC-MS system (RSLC, Thermo Fisher, Dreieich, Germany). The raw data was processed with MaxQuant software (version 1.5.3.30, Max-Planck Institute for Biochemistry, Computational Systems Biochemistry, Martinsried, Germany), and the peptides were searched against all human entries of the UniProtKB/Swiss-Prot database via the Andromeda search engine (Max-Planck Institute for Biochemistry, Computational Systems Biochemistry, Martinsried, Germany). A false discovery rate of 0.01 on the peptide and protein level was set. Data were analyzed in Perseus (version 1.5.2.6, Max-Planck Institute for Biochemistry, Computational Systems Biochemistry, Martinsried, Germany), and if applicable a two sided one-sample Student’s *t*-test was applied for comparison and was visualized via Graph Pad Prism (version 8, San Diego, CA, USA).

### 4.8. Bioinformatics Proteome Profiling Analysis

The workflow of the bioinformatics profiling approach is shown in Figure 3A. Proteins which showed significant deregulation (*p* < 0.05; logFC < –1/> 1) were analyzed for functional enrichment using g:Profiler web tool [44]. Selected significantly enriched processes were plotted using ggplot2 package version 3.3.0 [45] in R (version 3.6.3). In order to extend the functional insight, physical interaction partners from literature and databases were added to the 26 differentially expressed proteins using the Cytoscape (version 3.7.1) plugin Genemania version 3.5.1 [46]. Next, the Cytoscape plugin CyTargetLinker version 4.1.0 [47] was used to screen the interaction network for miR interaction partners using the mirTarBase [48]. The MCODE plugin version 1.5.1 [49] was used to identify highly interconnected functional clusters. Finally, miRs from the top three identified clusters were selected for further experimental analysis.

### 4.9. Statistical Analysis

The results were analyzed in Graph Pad Prism software (version 6 and 8, San Diego, CA, USA) and shown as means ± SEM. The statistical testing was performed via a two-tailed Student’s *t*-test for unpaired samples and a two-way ANOVA for a multiple group comparison. The statistical significance was set at *p* < 0.05.

## 5. Conclusions

We developed a comprehensive bioinformatics proteome profiling approach to track actively deregulated proteins in an ATG7-deficient molecular milieu and help identify its biologically relevant miR regulators. In addition to furthering the understanding of pathways involved with autophagy and senescence, our approach may be helpful in finding therapeutic strategies to counter aberrant autophagy in lung fibrosis. Notably, our bioinformatics screening approach enables a functional link from proteomics to non-coding RNAs, in which no further time- and cost-intensive high-throughput transcriptomic profiling method is needed. It not only investigates differentially expressed proteins and their function, but also relevant functional miR interaction partners. The approach is not limited to a specific cell-type and disease, thus highlighting its high relevance in proteome and non-coding RNA research.

## Supplementary Materials

Supplementary materials can be found at https://www.mdpi.com/1422-0067/21/11/4126/s1. Supplementary Table S1. Overview of the proteome dataset and the functional enrichment analysis using g: Profiler.
